# The XIth International Symposium on Thysanoptera and Tospoviruses Co-sponsored by Yunnan Academy of Agricultural Sciences and Nanjing Agricultural University in Kunming, China 2019

**DOI:** 10.3390/v11100939

**Published:** 2019-10-12

**Authors:** Xiaorong Tao, Zhongkai Zhang

**Affiliations:** 1 Department of Plant Pathology, Nanjing Agricultural University, Nanjing 210095, China; 2 Institute of Biotechnology and Genetic Resources, Yunnan Academy of Agricultural Sciences, Kunming 650223, China

The XIth International Symposium on Thysanoptera and Tospoviruses co-hosted by the Yunnan Academy of Agricultural Sciences, and Nanjing Agricultural University was held from September 21–25 in Kunming, China (Figure 1). The conference was jointly chaired by Professor Zhongkai Zhang from the Institute of Biotechnology and Genetic Resources of the Yunnan Academy of Agricultural Sciences and Professor Xiaorong Tao from the College of Plant Protection of Nanjing Agricultural University. More than 120 representatives from twelve countries, including the host country China, the United States, France, the Netherlands, Italy, the United Kingdom, Hungary, Australia, India, and Japan attended the conference. 

International Symposium on Thysanoptera and Tospoviruses is the most important professional conference in the field of thrips and tospoviruses, and is held every three to four years. Eighteen representatives, including academician Fang Rongxiang from the Chinese Academy of Sciences, Emeritus Professor Thomas L. German from the University of Wisconsin, Professor Diane E. Ullman from the University of California, Davis, Professor Stephane Blanc from the University of Montpellier, Professor Li Yi from Peking University, Professor Zhou Xueping from the Chinese Academy of Agricultural Sciences, Professor Taiyun Wei from the Fujian Agriculture and Forest University, and other colleagues gave plenary presentations (Figure 2). In addition, 37 representatives also gave presentations in the concurrent sessions on the topics of Molecular Biology of Tospoviruses, Biology and Systematics of Thrips, Virus–Vector–Host Interactions, Ecology and Behavior of Thrips, Virus Emergence, Genetics, and Diversity of Tospoviruses, Host Plant Resistance Against Tospoviruses and Thrips, and Integrated Management of Thrips and Tospoviruses (Figure 2).

The participants held heated discussions and in-depth exchanges on the latest research progress at the conference (Figure 3), which promoted researchers of different disciplines of thrips and tospoviruses to cooperate and to develop new strategies to control and manage thrips and tospoviruses diseases.

At the end of the program, two young colleagues from the next generation of tospoviruses and thrips researchers summarized highlights of this conference (Figure 4). Eight best talk awards from concurrent sessions and two best poster awards were selected by moderators and meeting committee members (Figure 4). The next International Symposium on Thysanoptera and Tospoviruses will be co-sponsored by the Wageningen University of the Netherlands and the Institute for Sustainable Plant Protection of Italy in Europe 2022 or 2023.

## Figures and Tables

**Figure 1:**
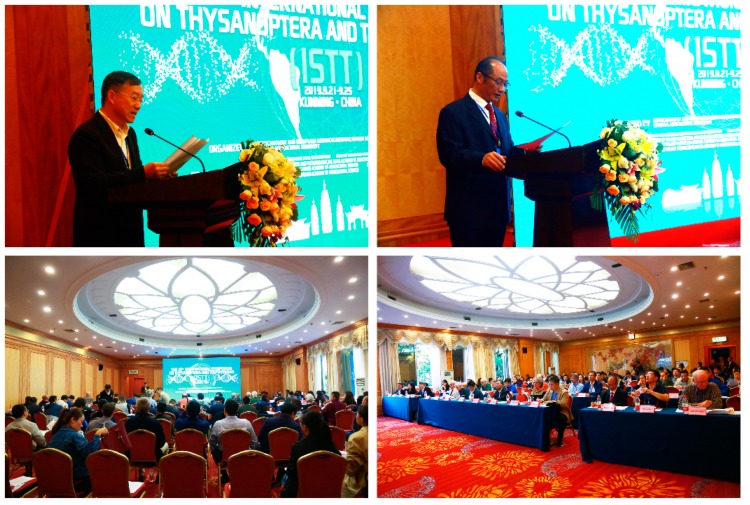
Opening ceremony of The XIth International Symposium on Thysanoptera and Tospoviruses in Kunming, China 2019.

**Figure 2:**
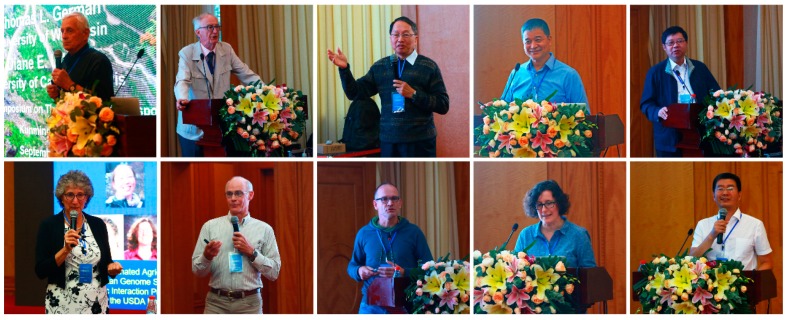
Representatives from all over the world gave presentations on the XIth International Symposium on Thysanoptera and Tospoviruses.

**Figure 3:**
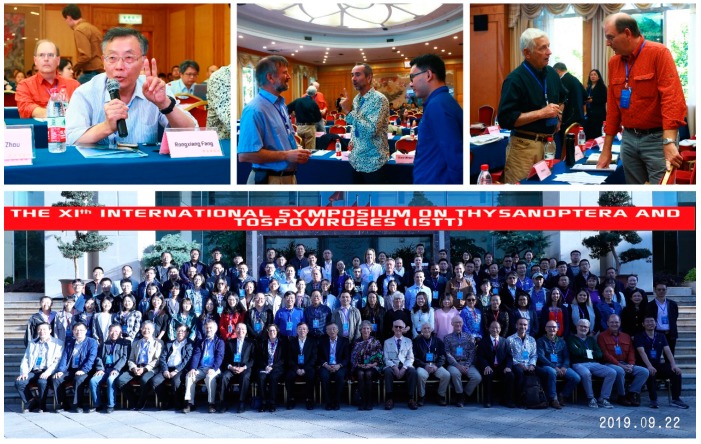
Group photo of representatives in the XIth International Symposium on Thysanoptera and Tospoviruses and heated discussions at the conference.

**Figure 4:**
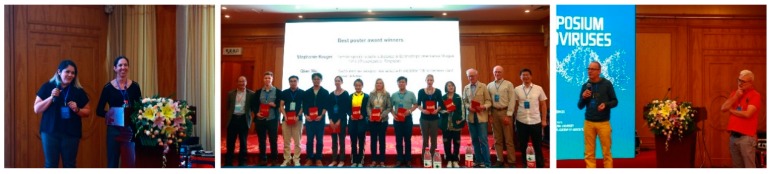
Highlights, best talk awards and best poster awards of this conference.

